# A Diffusion Model Analysis of Object-Based Selective Attention in the Eriksen Flanker Task

**DOI:** 10.1027/1618-3169/a000588

**Published:** 2023-09-29

**Authors:** Paul Kelber, Martina Gierlich, Jonathan Göth, Martin Georg Jeschke, Ian Grant Mackenzie, Victor Mittelstädt

**Affiliations:** ^1^ Department of Psychology, University of Tübingen, Germany

**Keywords:** Eriksen flanker task, object-based selective attention, attentional spreading, sensory enhancement, target attenuation, diffusion model for conflict tasks

## Abstract

**Abstract.** Selective attention might be space-, feature-, and/or object-based. Clear support for the involvement of an object-based mechanism is rather scarce, possibly because the predictions of models from these different classes often overlap. Yet, only object-based models can account for a larger congruency effect (CE) in the Eriksen flanker task when flankers are more (vs. less) strongly grouped to the target, but spacing and other response-irrelevant features of target and flankers are held constant. Exactly this was observed by Kramer and Jacobson (1991). So far, this theoretically relevant finding has not been replicated closely. We replicated the finding in two web-based experiments. Specifically, CEs were larger when flanker lines were connected to the central target line (vs. to outer neutral lines). We also successfully fitted the Diffusion Model for Conflict tasks (DMC) to the experimental data. Critically, diffusion modeling (DMC) and distributional analyses (delta functions) revealed that object membership primarily affected target processing strength rather than strength or timing of flanker processing. This challenges the prominent attentional spreading (sensory enhancement) account of object-based selective attention and motivates an alternative target attenuation account.


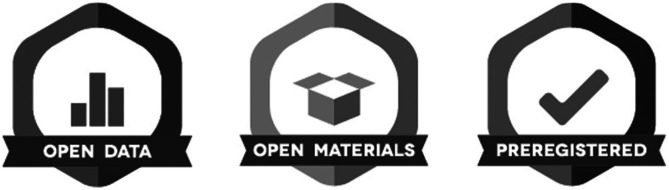


One major goal of cognitive psychology is to specify the mechanisms encompassing *selective attention*, the restriction of further processing to a subset of the available information. Many models of selective attention have been influenced by findings from the Eriksen flanker task (B. A. Eriksen & Eriksen, 1974). In this conflict task, participants are instructed to respond to a central target (e.g., left-hand and right-hand response to central letters S and H, respectively), which is accompanied by distractors (flankers) on each side. Target and flankers indicate the same response (congruent trials; e.g., SSS) or different responses (incongruent trials; e.g., HSH). A congruency effect (CE) – lower mean response time (RT) and/or higher proportion of correct responses (PC) in congruent compared to incongruent trials – has been observed across various flanker task settings (e.g., Fenske & Eastwood, 2003; Kopp et al., 1996; Rafal et al., 1996). As the size of the CE in the flanker task is (inversely) related to selective attention performance, models of selective attention should account for CE modulations.

A well-known CE modulation is its increase with decreasing spatial distance between target and flankers (e.g., B. A. Eriksen & Eriksen, 1974; Fox, 1998; Hommel, 1995; Miller, 1991; Schäfer & Frings, 2021; Servant & Evans, 2020; Shomstein & Yantis, 2002; St. James, 1990). First, this modulation is explained by space-based models, such as the spotlight model (Broadbent, 1982; Posner, 1980; Posner et al., 1980), the zoom lens model (C. W. Eriksen & St. James, 1986; C. W. Eriksen & Yeh, 1985), and the gradient model (Downing & Pinker, 1985). Differences aside, these models are united by the assumption that attentional processes operate on a still unstructured representation of external space and select entities based on their spatial position, leading to weaker selective attention (i.e., to a larger CE) for closer flankers.

Second, grouping-based models (e.g., Duncan, 1984; Egly et al., 1994; Kahneman & Henik, 1981; Neisser, 1967; Shomstein & Yantis, 2002; Vecera & Farah, 1994) can also account for the distance dependence of the CE in the flanker task. These models deny that attention is assigned to a contiguous and hitherto unparsed region within the visual field. Instead, they assume that attentional allocation is shaped by the perceptual grouping of elements in the preprocessed visual field. Accordingly, weaker selective attention for closer flankers is caused by stronger target–flanker binding (Gestalt principle of proximity; Wertheimer, 1923).

CE modulations by nonspatial factors are not readily explained by space-based models. By contrast, grouping-based models can account for a number of such CE modulations. For example, the CE has been shown to be larger when target and flankers are presented in the same color rather than in different colors (e.g., Harms & Bundesen, 1983). Distant flankers presented in the same color as the target have even been found to elicit a larger CE (Baylis & Driver, 1992) compared to close flankers presented in a different color, suggesting that the effect of common color (Gestalt principle of similarity) can override the effect of spatial proximity. In addition, Driver and Baylis (1989) observed a larger CE for distant flankers moving together with the target than for stationary flankers nearby the moving target. Accordingly, proximity can also be overridden by common motion (Gestalt principle of common fate).

Critically, grouping-based models may be subdivided into feature- and object-based models (for reviews, see Cavanagh et al., 2023; Chen, 2012; Maunsell & Treue, 2006). Feature-based models posit that when an entity with a specific feature value (e.g., blue) is attended, other entities are processed more strongly when their feature value is similar (vs. dissimilar). Assuming that target selection involves a preattentive analysis of features (e.g., color, motion direction) in the visual field, flankers similar (dissimilar) to the target should then have a larger (smaller) impact on target classification. While object-based models also assume a preattentive segmentation, they essentially posit that attention selects a full-blown object representation for further processing rather than a loosely connected set of similar entities. Thus, a standard assumption of object-based models is that attentional spread is stronger within an object than between objects (e.g., LaBerge & Brown, 1989; Roelfsema et al., 1998). Accordingly, attending to one part of an object strengthens the processing of the other parts of that object. Based on this, one would predict that CEs are enlarged when the flankers are grouped with the target because the flankers should then be processed more strongly.

However, none of the results in favor of grouping-based models described above clearly points to an object-based mechanism, since feature-based models can account for them as well. To assess the validity of object-based models, it is therefore vital to investigate whether the CE can be modulated by manipulating the grouping strength between target and flankers while keeping all response-irrelevant features of target and flankers constant. Following this approach, Kramer and Jacobson (1991; Experiment 3, *N* = 12) provided one of the strongest pieces of evidence to date for an object-based mechanism within the flanker task paradigm. Their participants had to indicate whether a central vertical line (target) was dotted or dashed (see Figure 1). To either side of the target, another vertical line (flanker) was presented. Flankers were either both dotted or both dashed. In addition to a distance manipulation, object membership of target and flankers was manipulated by connecting the flankers to the target (same-object condition) or to outer neutral lines (different-object condition). Thus, flankers were grouped with the target or with the outer lines by contour. Importantly, the CE (on both mean RTs and PCs) was affected not only by distance but also by object membership: It was larger for flankers near the target than for distant flankers, and it was larger in the same- (26 ms, 5.0%) than in the different-object condition (2 ms, 0.5%).^1^ Considering that it is not clear how either space- or feature-based models could account for the latter CE modulation, its observation points to the involvement of an object-based mechanism. However, in the absence of more fine-grained analyses than at the level of mean RTs and PCs, a more precise specification of the structure and the dynamics of the putative object-based mechanism seems out of reach.

**Figure 1:**
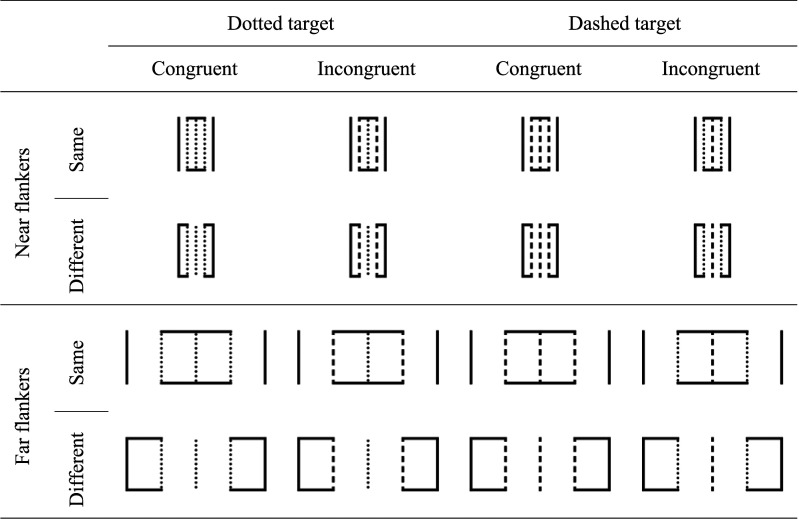
Illustration of the deployed stimulus material (not to scale).

One objective of the presented research was to examine whether the object-based CE modulation found by Kramer and Jacobson (1991) withstands replication in two pre-registered web-based experiments (*N* = 40 each). Although the robustness of the original finding has theoretical and also practical relevance (e.g., for human-like machine vision; Sun & Fisher, 2003), a close replication has not been reported yet to our knowledge. This seems unsatisfactory, especially given the mixed evidence regarding object-based selective attention. For instance, several attempts to replicate the superiority of common motion over proximity observed by Driver and Baylis (1989) have failed (Berry & Klein, 1993; Kramer et al., 1991). The superiority of common color over proximity observed by Baylis and Driver (1992) could also not be replicated (Fox, 1998; Moore et al., 2021). Therefore, we re-examined the unique prediction of object-based models that the CE (on mean RT and/or PC) is larger when target and flankers belong to the same as compared to different perceptual objects while keeping response-irrelevant features of target and flankers constant.

Another objective was to further elucidate the still elusive mechanism underlying object-based selective attention via diffusion model analyses. In general, CEs depend on the interplay of the relative strength and timing of target- and distractor-based response activation (e.g., Ulrich et al., 2015). Thus, any CE modulation (here, by object membership) could potentially reflect changes in multiple aspects of conflict processing, namely in target processing strength as well as in strength and/or speed of distractor processing. To address this issue, we fitted the *Diffusion Model for Conflict tasks* (DMC; Ulrich et al., 2015) to the experimental data. The DMC has been shown to account for the RT and PC distributions in various conflict task studies (e.g., Hübner & Töbel, 2019; Luo & Proctor, 2020; Mittelstädt et al., 2022; Servant & Evans, 2020; Ulrich et al., 2015) by relying on parameters that specify exactly how target and distractor processes interact over time. A more detailed description of the DMC is provided below in the dedicated Diffusion Modeling section.

In addition to diffusion model analyses, we also conducted fine-grained behavioral analyses of delta functions (De Jong et al., 1994), which illustrate how the CE size changes across the RT distribution. A comparison of delta function slopes between experimental conditions is informative for our purposes because these slopes are thought to reflect the timing of distractor-based response activation (e.g., Burle et al., 2005; Ellinghaus & Miller, 2018; Hübner & Töbel, 2019; Mackenzie et al., 2022; Ulrich et al., 2015). Thus, as will be discussed in more detail later, these diffusion model and distributional analyses allow a closer specification of the suspected object-based mechanism (e.g., flanker enhancement vs. target attenuation).

## Experiment 1

Experiment 1 followed Kramer and Jacobson (1991; Experiment 3) closely. The factors *congruency* (congruent vs. incongruent), *proximity* (near vs. far), and *object membership* (same vs. different) were manipulated within subjects and blocks. The critical focus was to investigate whether CEs are modulated not only by proximity but also by object membership, which is predicted only by object-based models (and not by space- or feature-based models).

### Method

#### Participants

Following our pre-registration, 40 participants (30 female, 36 right-handed, *M*_age_: 23.1, age range: 18–54 years) were tested online. The sample size was determined based on our available resources. It gave us a statistical power of at least 1 − β = .80 for detecting a small-to-medium effect size of *f* = 0.23 in a repeated-measures ANOVA with α = .05. All participants reported German language proficiency and normal or corrected-to-normal vision. They were recruited from the student pool at the University of Tübingen, provided informed consent prior to the experiment, and could receive course credit in exchange for taking part in one session, which lasted about 30 min.

#### Apparatus and Stimuli

Stimulus presentation and response recording were controlled by *jsPsych* (de Leeuw, 2015). The experiment was hosted on a website of the University of Tübingen and could be started via various browsers on the participants’ computers (minimum screen resolution: 960 *×* 720 pixels). To render the stimulus size comparable across devices, a calibration routine was included at the start of the experiment in which participants were requested to resize a rectangular area on their screen to the size of a physical bank card or ID card. All visual stimuli were presented in black on a white background. Figure 1 illustrates the 16 possible stimulus displays (two target line types × two flanker line types × two distances × two object membership divisions). Each display consisted of five parallel vertical lines. The central vertical line (dotted or dashed) served as the target and the inner peripheral vertical lines (dotted or dashed) as flankers. Target and flanker lines matched (mismatched) in congruent (incongruent) trials. Object membership was manipulated by connecting the flankers to the target (same object) or to the outer peripheral solid vertical lines (different object). Dotted and dashed target lines were randomly mapped to the response keys “Q” and “P” for each participant.

#### Procedure

Participants were tested in one practice and 14 experimental blocks. Each block comprised 64 randomly ordered trials (four repetitions of 16 stimulus displays). In the beginning, participants were instructed to classify the central line as quickly and accurately as possible. This instruction was provided again after each block together with performance feedback (mean RT and PC). Each trial began with the presentation of a central fixation cross (500 ms). Following its offset, the line array was presented in the screen center until participants responded (up to a maximum of 2 s). If necessary, visual feedback (2 s) indicated that the response was incorrect, too slow (not given within 2 s), or too fast (< 150 ms). The trial ended with a blank screen (500 ms).

### Results and Discussion

**Figure 2:**
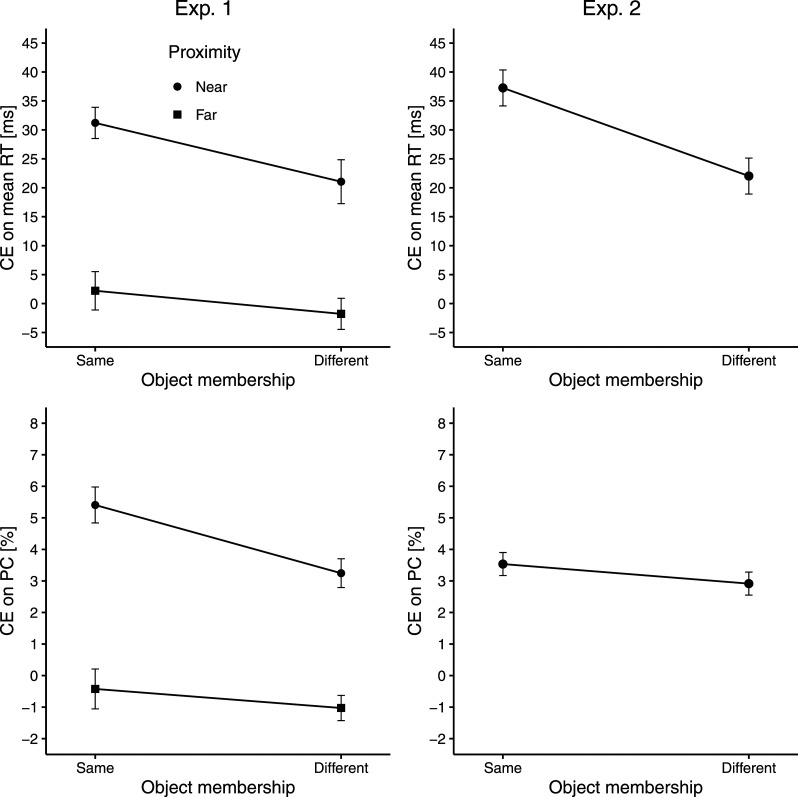
CE on mean RT (top) and PC (down) from Experiment 1 (left) and Experiment 2 (right) as a function of proximity (near vs. far; Experiment 1 only) and object membership (same vs. different). Circles and squares represent the mean across participants. Error bars represent ± 1 within-subjects standard error of the mean according to the method by Cousineau (2005) with the correction from Morey (2008).

The practice block and responses considered too slow (0.1%) or too fast (0.02%) were excluded from all analyses. Choice errors (5.1%) were additionally excluded from RT analyses. Figure 2 (left) depicts the CEs on mean RT (top) and PC (bottom) obtained in Experiment 1. Mean RTs and PCs (see Table 1) were subjected to separate three-way ANOVAs with repeated measures (*Congruency × Proximity × Object membership*).

**Table 1:** Mean RT and PC in Experiment 1 and Experiment 2 as a function of proximity (near vs. far; Experiment 1 only), object membership (same vs. different), and congruency (congruent vs. incongruent)

Experiment	Proximity	Object membership	Congruency	Mean RT (ms)	PC (%)
1	Near	Same	Congruent	532 (2)	96.6 (0.3)
	Near	Same	Incongruent	563 (3)	91.2 (0.7)
	Near	Different	Congruent	527 (3)	96.8 (0.4)
	Near	Different	Incongruent	548 (3)	93.5 (0.4)
	Far	Same	Congruent	517 (2)	96.0 (0.4)
	Far	Same	Incongruent	520 (3)	96.4 (0.4)
	Far	Different	Congruent	527 (2)	93.9 (0.4)
	Far	Different	Incongruent	525 (2)	94.9 (0.4)
2	Near	Same	Congruent	527 (2)	96.8 (0.4)
	Near	Same	Incongruent	565 (3)	93.2 (0.4)
	Near	Different	Congruent	522 (2)	96.7 (0.4)
	Near	Different	Incongruent	545 (2)	93.8 (0.5)
*Note*. Within-subjects standard errors of the mean are provided in parentheses.					

For mean RTs, the main effect of *congruency* was significant, *F*(1, 39) = 55.72, *p < .*001, η_*p*_^2^ = .59, reflecting a CE (539 − 526 = 13 ms). The main effect of *proximity* was significant as well, *F*(1, 39) = 47.98, *p < .*001, η_*p*_^2^ = .55, indicating slower responses in the near (542 ms) than in the far condition (522 ms). There was also a significant interaction between *congruency* and *proximity*, *F*(1, 39) = 46.08, *p < .*001, η_*p*_^2^ = .54, which was driven by a larger CE in the near (26 ms) than in the far condition (0 ms).^2^ Critically, the interaction between *congruency* and *object membership* was also significant, *F*(1, 39) = 6.18, *p* = .017, η_*p*_^2^ = .14. In line with object-based models, the CE was larger in the same- (17 ms) than in the different-object condition (10 ms).^3^ Finally, there was a significant interaction between *proximity* and *object membership*, *F*(1, 39) = 33.66, *p < .*001, η_*p*_^2^ = .46. For near flankers, responses were significantly slower in the same- (547 ms) than in the different-object condition (538 ms), *t*(39) = 4.87, *p < .*001, *d_z_* = 0.77. For far flankers, in contrast, responses were significantly slower in the different- (526 ms) than in the same-object condition (518 ms), *t*(39) = 3.66, *p < .*001, *d_z_* = 0.58. No other effect in the three-way ANOVA was significant (all *p*s *> .*256).

The PC pattern paralleled the mean RT pattern. There were again significant main effects of *congruency* (CE = 95.8 − 94.0 = 1.8%), *F*(1, 39) = 20.11, *p < .*001, η_*p*_^2^ = .34, and *proximity* (near: 94.5%, far: 95.3%), *F*(1, 39) = 4.17, *p* = .048, η_*p*_^2^ = .10. Furthermore, there was a significant interaction between *congruency* and *proximity*, *F*(1, 39) = 66.52, *p < .*001, η_*p*_^2^ = .63, indicating a larger CE for near (4.3%) than for far flankers (−0.7%). Importantly, the interaction between *congruency* and *object membership* was also significant, *F*(1, 39) = 7.64, *p* = .009, η_*p*_^2^ = .16. The CE was larger in the same- (2.5%) than in the different-object condition (1.1%). Finally, *object membership* and *proximity* interacted, *F*(1, 39) = 25.50, *p < .*001, η_*p*_^2^ = .40. For near flankers, responses were significantly less accurate in the same- (93.9%) than in the different-object condition (95.2%), *t*(39) = 3.46, *p* = .001, *d_z_* = 0.55, whereas for far flankers, responses were significantly less accurate in the different- (94.4%) than in the same-object condition (96.2%), *t*(39) = 4.10, *p < .*001, *d_z_* = 0.65. No other effect was significant (all *p*s *> .*077).

## Experiment 2

As observed by Kramer and Jacobson (1991) and predicted by object-based models, the CE was larger in Experiment 1 when flankers were connected to the target (vs. to outer lines). The goal of Experiment 2 was to replicate the critical findings from Experiment 1 (including the diffusion modeling results; see below). Experiment 2 was identical to Experiment 1 except that we only manipulated *congruency* (congruent vs. incongruent) and *object membership* (same vs. different) for near flankers. We removed the distance manipulation from Experiment 2 for two reasons. First, CEs were absent with far flankers in Experiment 1, relieving us from the need to account for a failure of selective attention in the far conditions. Second, any result based on a spatial distance manipulation would remain dubious in an online study, in which viewing distance, and thus perceived target-flanker distance, is not controllable.

### Method

#### Participants

Forty participants from the same participant pool were tested online. Data from two participants were excluded because their overall PC (72.7% and 0.0% due to nonresponding) was below 75%. The age of the remaining 38 participants (28 female, 34 right-handed) ranged from 19 to 54 years (*M* = 23.1).

#### Apparatus, Stimuli, and Procedure

Everything was identical to Experiment 1 except that only the eight stimulus displays with near flankers were used. Thus, participants were tested in one practice and only seven experimental blocks with 64 trials each (eight repetitions of eight stimulus displays).

### Results and Discussion

In total, 0.1% of the responses were too slow, 0.02% too fast, and 4.9% incorrect. Figure 2 (right) visualizes CEs on mean RT (top) and PC (bottom) in Experiment 2. Mean RTs and PCs (see Table 1) were subjected to separate two-way ANOVAs with repeated measures (*Congruency × Object membership*). For mean RTs, there were significant main effects of *congruency* (CE = 555 − 525 = 30 ms), *F*(1, 37) = 131.45, *p < .*001, η_*p*_^2^ = .78, and *object membership* (same: 546 ms, different: 533 ms), *F*(1, 37) = 24.58, *p < .*001, η_*p*_^2^ = .40. Crucially, the interaction between *congruency* and *object membership* was significant, *F*(1, 37) = 11.98, *p* = .001, η_*p*_^2^ = .24. This indicates that the CE was again larger in the same- (37 ms) than in the different-object condition (22 ms).

For PCs, only the main effect of *congruency* was significant (CE = 96.7 − 93.5 = 3.2%), *F*(1, 37) = 34.26, *p < .*001, η_*p*_^2^ = .48 (all other *p*s > .236). Although the interaction was not significant, the CE was numerically larger in the same- (3.5%) than in the different-object condition (2.9%), ruling out a speed–accuracy tradeoff.

## Distributional Analyses

The empirical results of both experiments indicate that mean CEs were smaller when target and flankers belonged to different perceptual objects (vs. to the same) although all response-irrelevant features of target and flankers were held constant. This replicates the finding by Kramer and Jacobson (1991) in an online setting and thus provides further support for the involvement of an object-based mechanism in selective attention. In a next step, we conducted additional distributional analyses to better understand the causes driving the object-based CE modulation. Specifically, RT delta functions were constructed separately for each participant and each condition (Experiment 1: near-same, near-different, far-same, far-different; Experiment 2: near-same, near-different).^4^ To create the RT delta functions, the nine deciles (0.1, 0.2, …, 0.9) of the RT distribution were computed separately for incongruent and congruent trials. Thus, the RT delta functions illustrate the difference between corresponding deciles (*RT_incongruent_* − *RT_congruent_*) as a function of response speed and are thought to reflect the time course of distractor-based activation (e.g., Burle et al., 2005; Ellinghaus & Miller, 2018; Hübner & Töbel, 2019; Mackenzie et al., 2022; Ulrich et al., 2015). For completeness, delta functions were also constructed for PCs based on CEs (*PC_congruent_* − *PC_incongruent_*) within five RT bins (0–20, 20–40, …, 80–100%).

In studies using the Eriksen flanker task, RT delta functions are typically increasing, reflecting larger CEs for slower responses. Considering that responses in the same-object condition were generally slower than in the different-object condition, the larger CE could be solely due to flanker-based activation having more time to develop before being superimposed with target-based activation. For example, it is possible that the object manipulation primarily affected the speed of early sensory processes rather than aspects of the response selection process involved in conflict processing (such as the strength of target and/or flanker processing). If this was the case, we should observe that RT delta functions for the object conditions overlap, indicating that, for the same RT, the CE is similar across conditions (for examples of overlapping delta functions for conditions that vary in response speed, see Mittelstädt et al., 2022).

**Figure 3:**
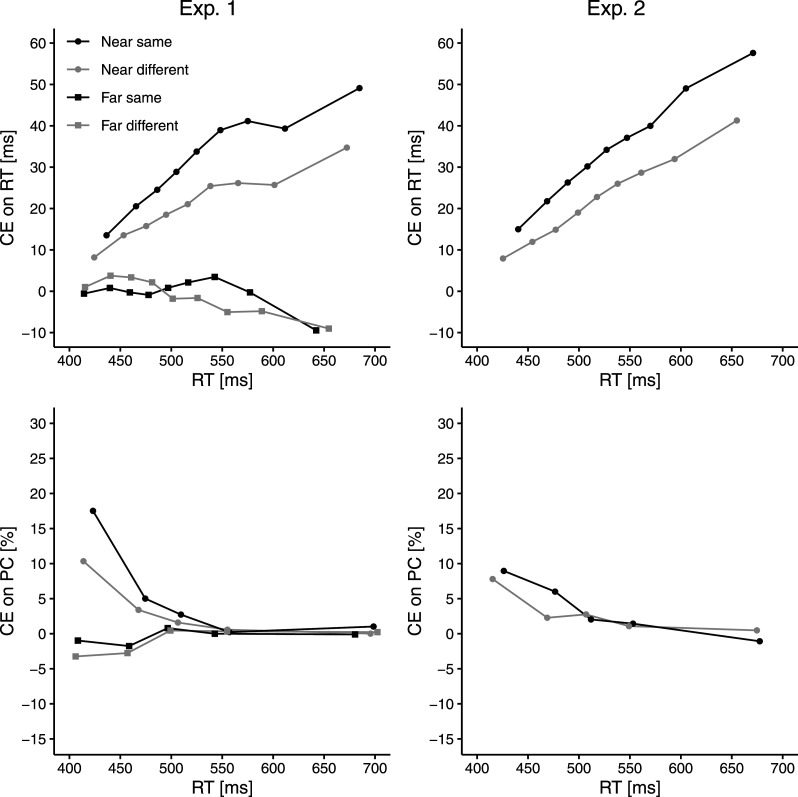
Delta functions for RTs (top) and PCs (bottom) in Experiment 1 (left) and Experiment 2 (right) as a function of proximity (near vs. far; Experiment 1 only) and object membership (same vs. different).

Figure 3 illustrates delta functions for RTs (top) and PCs (bottom) from Experiment 1 (left) and Experiment 2 (right). Visual inspection of the PC delta functions reveals the typical pattern that CEs on PC were largest for the fastest responses and declined for slower responses (see, e.g., Ellinghaus & Miller, 2018; Mittelstädt, Ulrich, et al., 2023; Ulrich et al., 2015). Consistent with previous flanker task studies, the RT delta functions in the near conditions were always increasing. Importantly, RT delta functions in the (near-)same condition appear to be shifted upward along the *y*-axis compared to the (near-)different condition, indicating that the CE was increased in the same-object (vs. different-object) condition across the entire range of RTs. This suggests that the object-based CE modulation was due to changes in conflict processing rather than due to a combination of slowing in the same-object (vs. different-object) condition and generally increasing flanker-based activation over time.

Another notable finding is that the RT delta functions in the two object conditions followed similar time courses, that is, they were approximately parallel. Previous studies have suggested that less steeply increasing or more strongly decreasing RT delta function slopes might reflect faster suppression of distractor-based activation (e.g., Ridderinkhof, 2002). Therefore, the approximately parallel offset of the RT delta functions across object conditions suggests that the relative strength of target-to-distractor activation, rather than its timing, was influenced by the object manipulation.

The statistical tests reported below support the interpretation based on visual inspection. Specifically, a line was fitted to the CEs at the nine deciles, whose intercept represents the predicted CE at the participant’s mean RT across conditions (e.g., Ellinghaus & Miller, 2018; Gade et al., 2020; Hübner & Töbel, 2019; Mittelstädt & Miller, 2018, 2020; Pratte et al., 2010). In both Experiment 1 and Experiment 2, the object manipulation significantly affected the intercepts, but not the slopes.^5^

### Experiment 1

RT delta function slopes and intercepts were subjected to separate two-way ANOVAs with repeated measures (*Proximity × Object membership*). For the intercepts, significant main effects of *proximity*, *F*(1, 39) = 58.04, *p < .*001, η_*p*_^2^ = .60, and *object membership*, *F*(1, 39) = 5.91, *p* = .020, η_*p*_^2^ = .13, indicate that, even when controlling for RT, predicted CEs were larger for near (25 ms) than for far flankers (−2 ms) and larger for the same-object (14 ms) than for the different-object condition (9 ms). The object-based modulation of the predicted CE was significant for near flankers (same: 29 ms, different: 20 ms), *t*(39) = 2.78, *p* = .008, *d_z_* = 0.44, but not for far flankers (same: −1 ms, different: −2 ms), *t*(39) = 0.18, *p* = .859, *d_z_* = 0.03. However, the two-way interaction between *proximity* and *object membership* was only marginally significant, *F*(1, 39) = 3.10, *p* = .086, η_*p*_^2^ = .07.

For the slopes, by contrast, there was only a significant main effect of *proximity*, *F*(1, 39) = 36.30, *p < .*001, η_*p*_^2^ = .48, showing that CEs are rising steeper for near flankers (0.15) compared to far flankers (−0.04). No other effect was significant (both *p*s > .331). Notably, paired *t*-tests indicated that delta function slopes did not differ significantly between the near-same (.16) and the near-different condition (.14), *t*(39) = 0.43, *p* = .667, *d_z_* = 0.07, nor between the far-same condition (−0.02) and the far-different condition (−0.06), *t*(39) = 1.05, *p* = .302, *d_z_* = 0.17. Taken together, the object-based CE modulation can be attributed to differential conflict processing (as reflected in a vertical shift), but rather not to differential distractor processing speed (since parallelity was not systematically violated).

### Experiment 2

Slopes and intercepts were subjected to separate paired *t*-tests. As in Experiment 1, intercepts were significantly larger in the same-object (33 ms) than in the different-object condition (23 ms), *t*(37) = 2.67, *p* = .011, *d_z_* = 0.43, whereas slopes did not differ significantly between the same-object condition (.18) and the different-object condition (.15), *t*(37) = 0.76, *p* = .453, *d_z_* = 0.12. This provides further evidence that the object-based CE modulation is reflected in differential conflict processing, but rather not in differential distractor processing speed.

## Diffusion Modeling

The results of Experiment 1 and Experiment 2 were consistent with the prediction of object-based models that the mean CE should be larger when flankers are grouped with the target (vs. with neutral items). Furthermore, the distributional analyses suggest that the object manipulation affected conflict-related processes rather than just the speed of conflict-unrelated processes. However, conflict processing (as well as thus the CE size) depends on the interplay of the strength and timing of target relative to distractor (here, flanker) processing. It is thus still unclear whether the larger CE for stronger target–flanker grouping resulted from changes in target processing strength (i.e., target attenuation), distractor processing strength (i.e., distractor enhancement), and/or distractor processing timing. Assuming that RT delta function slopes reflect the speed of distractor processing, it seems that the processes underlying object-based selective attention primarily altered the strength of target-to-distractor processing, since the RT delta functions followed statistically indistinguishable time courses in the same- and different-object conditions.

But the above interpretation of delta function slopes is not without alternatives (W. Schwarz & Miller, 2012). Even more importantly, analyses of delta function slopes are not capable of dissociating distractor enhancement from target attenuation. To address these issues, we fitted several versions of the DMC (Ulrich et al., 2015) to the experimental data in the near conditions. The central assumption of the DMC is that response activation is the result of the superposition of target- and distractor-based activation in a single Wiener diffusion process heading toward the correct decision boundary (with diffusion constant σ). The drift rate of this superimposed process at each time point *t* is given by the sum of the time-invariant input from the target-based process with drift rate μ*_t_* and the time-variant input from the distractor-based process with drift rate μ*_d_*(*t*). Specifically, the input from the distractor-based process is modeled by a scaled Gamma density function with shape parameter *a*, which raises to a single maximum *A* at time *t_peak_* = (*a* − 1) τ and then fades out to zero. RT then amounts to the sum of the time needed for reaching a decision boundary and the time needed for normally distributed nondecision (residual) processes (with μ*_r_* and σ*_r_*). Starting point variability is implemented by a starting point distribution with shape parameter *α_s_*.

Our analysis focused on the influence of object membership on the three DMC parameters μ*_t_* (target processing strength), *A* (distractor processing strength), and τ (distractor processing timing). These parameters describe the interplay of target- and distractor-based activation in conflict processing, which seems to be influenced by the object manipulation according to the distributional analyses reported above.

### Fitting Details

Eight models were fitted to the data in each experiment. In all models, three parameters were estimated but were not allowed to vary between the same- and different-object conditions, namely mean residual time (μ*_r_*), *SD* of residual time (σ*_r_*), and correct decision boundary (*b*), because they cannot account for the observed object-based CE modulation: Residual processes equally affect performance in congruent and incongruent trials (thus ruling out μ*_r_* and σ*_r_*), and criterion shifts should become apparent in speed–accuracy tradeoffs, which the object-based CE modulation was not prone to (thus ruling out *b*). Theoretically, it therefore seemed plausible to assume that only combinations of the three conflict-related parameters μ*_t_*, *A*, and τ can vary between the same- and different-object conditions within each participant. As a result, one model (*M*_*μ_t_*,*A*,*τ*_) allowed the variation of all three parameters, three models (*M*_*μ_t_*,*A*_, *M*_*μ_t_*,*τ*_, *M*_*A*,*τ*_) allowed two parameters to vary, three more models allowed only one parameter to vary (*M*_*μ_t_*_, *M_A_*, *M*_*τ*_), and – as a baseline – one model (*M*_ø_) allowed the variation of neither parameter.

These eight models were fitted to the data in both congruent and incongruent trials separately for each participant and each of the two near conditions (same- vs. different-object) using the *R* package *DMCfun* (Mackenzie & Dudschig, 2021). The diffusion constant was fixed to σ = 4, the shape parameter of the starting point distribution to *α_s_* = 3, and the shape parameter of distractor-based activation to *a* = 2 (see, e.g., Ellinghaus et al., 2018; Ulrich et al., 2015). For *a* = 2, τ can be identified with the time of the peak amplitude of distractor-based activation *t_peak_* (i.e., *t_peak_* = τ (*a* − 1) = τ). The default values of the *R* function *dmcFitSubjectDE* from the package *DMCfun* (version 3.4.0; Mackenzie & Dudschig, 2021) were used to describe the parameter space with the following exceptions: To allow *A* and τ to cover a larger parameter space, the minimum *A* (τ) was lowered to 0 (1 ms) and the maximum τ was raised to 500 ms; in addition, the maximum *b* was set to 100, the maximum μ*_r_* to 500 ms, and the maximum σ*_r_* to 50 ms.

Following Servant and Evans (2020), parameter values were estimated by minimizing the likelihood-ratio chi-square statistic *G*^2^ that describes the goodness of fit of the predicted and observed cumulative distribution functions (five percentiles) and conditional accuracy function (five bins). To this end, a differential evolution optimization algorithm (see Mackenzie & Dudschig, 2021; Ulrich et al., 2015) was carried out in 10,000 simulated trials per condition. The parameter search was terminated either when the last 50 iterations did not reduce *G*^2^ by at least 1% or at the latest after 500 iterations. As a metric of the balance between goodness of fit (*G*^2^) and complexity (number of free parameters), the *Bayesian information criterion* (BIC; G. Schwarz, 1978) was computed (see Servant & Evans, 2020). The lower the BIC value, the better the model.

### Fitting Results

**Figure 4:**
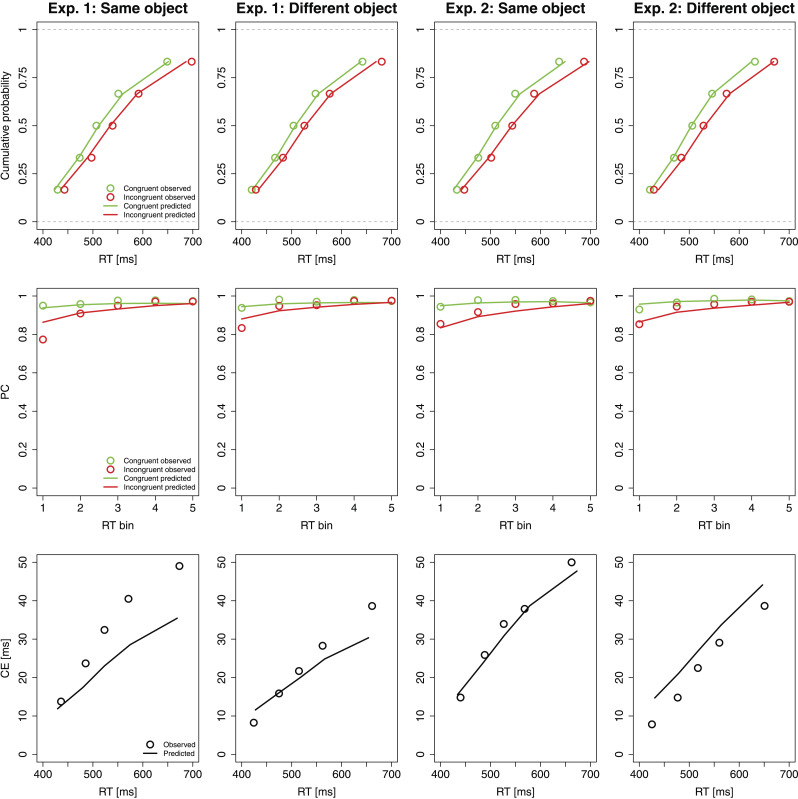
Cumulative distribution function (first row), conditional accuracy function (second row), and RT delta function (third row) for the (near-)same and (near-)different object condition observed in Experiment 1 and Experiment 2 and predicted by *M*_*μ_t_*_.

Table 2 contains the best-fitting parameter values (means across participants) and the BIC values (means across participants and conditions) for each of the eight models in each experiment. In general, BIC values of the models in the present study were comparable to a previous application by Servant and Evans (2020), in which the number of free parameters and experimental trials was similar. For both experiments in the present study, *M*_*μ*_*t*__ had the lowest BIC value and thus proved to be the best model. As can be seen in Figure 4, this rather simple model already provided a good fit to the distributional RT and PC data. Accordingly, the comparison of BIC values across the hierarchy of models suggests that only μ*_t_* varied between the same-object and different-object conditions.

**Table 2:** Mean best-fitting values for the DMC parameters μ_*t*_, *A*, τ, *b*, μ*_r_*, and σ*_r_* for each model ordered by ascending BIC value for each experiment

Experiment	Model	μ*_t_*	*A*	τ	*b*	μ*_r_*	σ*_r_*	BIC
1	*M*_*μ*_*t*__	0.58, 0.62 (.022)	13.8	249	74.5	408	38	111.1
	*M_Ø_*	0.57	14.0	217	73.6	408	36	112.3
	*M*_*τ*_	0.60	17.0	243, 229 (.716)	76.2	409	37	113.9
	*M_A_*	0.58	16.0, 15.1 (.630)	226	74.3	409	38	114.1
	*M*_*μ*_*t*_,__*τ*_	0.55, 0.59 (.028)	15.7	237, 243 (.876)	71.8	409	37	114.1
	*M*_*μ*_*t*_,*A*_	0.55, 0.59 (.018)	16.8, 14.8 (.269)	227	72.7	409	36	116.1
	*M*_*μ*_*t*_,*A*,*τ*_	0.54, 0.59 (.007)	17.9, 15.6 (.271)	218, 242 (.488)	71.5	409	37	117.2
	*M_A_*,_*τ*_	0.59	15.2, 14.7 (.816)	240, 206 (.385)	74.8	407	37	117.5
2	*M*_*μ*_*t*__	0.58, 0.64 (.010)	17.8	254	72.8	413	38	121.1
	*M*_*μ*_*t*_,*τ*_	0.55, 0.62 (< .001)	17.6	257, 244 (.726)	69.0	415	39	124.1
	*M*_*μ*_*t*_,*A*_	0.56, 0.63 (.005)	22.1, 18.3 (.145)	269	69.3	416	40	125.5
	*M_Ø_*	0.61	19.0	275	72.0	415	39	125.9
	*M*_*μ*_*t*_,*A*,*τ*_	0.54, 0.63 (<.001)	20.0, 17.2 (.229)	252, 224 (.433)	70.8	414	39	126.1
	*M*_*τ*_	0.59	20.4	268, 230 (.335)	71.4	416	40	126.2
	*M_A_*,_*τ*_	0.60	18.6, 20.6 (.393)	259, 221 (.288)	72.6	414	38	127.3
	*M_A_*	0.60	19.1, 18.2 (.711)	234	71.5	417	40	130.3
*Note*. If two values are listed, the first refers to the (near-)same condition, the second to the (near-)different condition, and the value in brackets to the *p*-value of a paired *t*-test of the individual best-fitting values. BIC values are means across the (near-)same and the (near-)different condition and across participants.								

However, this does not entail that μ*_t_* varied *systematically* across participants. Similarly, *A* and/or τ might vary systematically according to other models, whose mean BIC values were, after all, only slightly inferior.

We thus performed paired *t*-tests for the best-fitting parameter values in the two object conditions for each model and each parameter that was allowed to vary between conditions. The resulting *p*-values are also reported in Table 2. Consistently across models and experiments, μ*t* was significantly lower in the same-object condition than in the different-object condition (all *p*s < .029). By contrast, *A* and τ did not differ significantly between the two object conditions in any model (all *p*s > .144).

One potential reason for the failure to find significant differences for *A* and τ might have been that these parameters are not estimated accurately. Indeed, White et al. (2018) showed that DMC parameter recovery was better for μ*_t_* than for *A* and τ, but generally improved with the number of simulated trials (50–5,000). Therefore, we fitted μ_*t*_, *A*, and τ (i.e., *M*_*μ*_*t*__, *A*, *τ*) again to the data in Experiment 1 and Experiment 2, but this time with 100,000 simulated trials. Once more, the effect on *A* and τ was not significant; if anything, it further diminished. Specifically, in Experiment 1, *object membership* significantly affected μ*_t_* (same: 0.52, different: 0.56), *t*(39) = 2.73, *p* = .010, *d_z_* = 0.43, but neither *A* (same: 16.5, different: 17.7), *t*(39) = 0.65, *p* = .518, *d_z_* = 0.10, nor τ (same: 182 ms, different: 202 ms), *t*(39) = 0.49, *p* = .630, *d_z_* = 0.08. Similarly, in Experiment 2, μ*_t_* differed significantly between object conditions (same: 0.53, different: 0.60), *t*(37) = 3.77, *p < .*001, *d_z_* = 0.61, whereas *A* (same: 20.6, different: 20.7), *t*(37) = 0.04, *p* = .970, *d_z_* = 0.01, and τ (same: 235 ms, different: 244 ms), *t*(37) = 0.26, *p* = .795, *d_z_* = 0.04, did not.

Taken together, the modeling results consistently indicate that the object manipulation led to an attenuated target processing strength in the same-object (vs. different-object) condition, whereas we could not find evidence for a reflection in distractor processing strength or timing.^6^

## General Discussion

In two online experiments reminiscent of Kramer and Jacobson (1991; Experiment 3), CEs were more pronounced when flankers belonged to the same perceptual object as the target (vs. to different ones). The present study thus provides direct evidence that human selective attention has an object-based facet. This seems particularly instructive given that other cornerstone discoveries in favor of object-based models (Baylis & Driver, 1992; Driver & Baylis, 1989) could not be replicated (Berry & Klein, 1993; Fox, 1998; Kramer et al., 1991; Moore et al., 2021) while other findings (e.g., Harms & Bundesen, 1983) can also be explained by feature-based models. Moreover, diffusion model analyses suggest that this object-based component of selective attention primarily relates to target processing strength. Specifically, the DMC fitting results consistently revealed smaller drift rates of target-based evidence accumulation when the target was perceptually bound to the flankers than when it was perceptually detached. In contrast, the DMC parameters that reflect strength and timing of distractor-based activation were not reliably affected by object membership.

The results of the distributional analyses agree well with those of the diffusion model analyses. Delta function slopes, which appear to be indicative of distractor-based activation timing, did not deviate systematically between object conditions in either experiment. This is in line with the fact that the DMC timing parameter τ was also not reliably affected by object membership. In addition, delta functions in the same-object (vs. different-object) condition were shifted upward along the *y*-axis. The fact that CEs were larger in the same- than in the different-object condition throughout the RT distribution illustrates that the object-based CE modulation observed at the mean RT level was actually conflict-related (i.e., due to target attenuation and/or flanker enhancement) and not just a by-product of speed differences between the object conditions (see Mittelstädt et al., 2022). Because CEs tended to be larger for slower responses (as reflected in positive-going delta functions), the same mean RT pattern could have also arisen due to slowing of conflict-unrelated processes in the same-object (vs. different-object) condition or due to a strategic criterion shift (i.e., more cautious responding in the same- than in the different-object condition). By ruling out such possibilities, our distributional analysis highlights the utility of analyzing data beyond the mean RT level. Consistent with our results, changes in drift rate should shift the delta functions up and down according to DMC simulations reported by Mittelstädt and Miller (2018). Furthermore, our simulations also confirmed that a reduction in drift rate alone can enlarge CE modulations at the mean RT level if distractor-based activation peaks rather late (such as in the flanker task used here). Taken together, our diffusion modeling and distributional analyses at least suggest that the reflection of object membership in distractor processing, if any, is minuscule compared to its strong and stable reflection in target processing.

The present findings allow us to discriminate between several accounts of object-based selective attention. The attentional spreading (or sensory enhancement) account (e.g., LaBerge & Brown, 1989; Roelfsema et al., 1998) asserts that allocation of attentional resources (1) adheres to object boundaries and (2) improves the quality of the respective perceptual representation. Put differently, attentional spread is expected to be stronger within objects than between objects, leading to enhanced processing of items in the same (vs. in a different) object. In this sense, one object is selected for further processing. Desimone and Duncan (1995) described how the human nervous system could realize this object selection process as a competition between object representations for scarce processing resources, which is biased bottom-up by perceptual segmentation (e.g., figure-ground separation) and top-down by the relevance for the current task (e.g., target classification). Attentional spreading predicts an object-based CE modulation due to flanker enhancement (as reflected in a larger amplitude of distractor-based activation) in the same-object (vs. different-object) condition. This prediction is inconsistent with the present modeling results. Alternatively, one could assume that it is not the maximum of the flanker representation quality but its timing that differs between object conditions. Specifically, distractor-based activation may last longer in the same-object (vs. different-object) condition. This is also not supported by our modeling and distributional analyses.^7^

Unlike the other object-based accounts presented above, a target attenuation account can explain the effects of object membership found in the behavioral data and in the best-fitting DMC parameters. When the target is grouped with other items (as opposed to ungrouped), it is less salient, so its processing strength is reduced, which in turn increases the relative contribution of flanker-based activation to the response selection process (without affecting flanker processing itself). Similarly, a number of manipulations that should decrease target processing strength (e.g., smaller or less intense targets) have been found to increase the CE in the Eriksen flanker task (e.g., C. W. Eriksen & Schultz, 1979; but see Servant et al., 2014). Hence, the decisive factor of the object manipulation is not whether the flankers are grouped with the target but whether the target is grouped with other items at all. Target classification is less laborious and therefore less susceptible to activation spilling over from flanker processing when the target is perceptually detached (in the different-object condition) than when it is subordinate to a higher-level perceptual object (in the same-object condition). No such difference should have occurred in flanker processing, since flankers were grouped in both the same- and different-object conditions (either with the target or the outer lines).

By rejecting a flanker-centered explanation, the target attenuation account follows the image segmentation hypothesis recently put forward by Moore et al. (2021) according to which grouping effects in the flanker task are not due to the selection of a perceptual group but to a difference in image quality. Specifically, feature discontinuities present in many different-object conditions are assumed to facilitate image segmentation in task-relevant and task-irrelevant parts. It is, however, not entirely clear how the image segmentation hypothesis could account for our modeling result that only target processing differs markedly between object conditions. Rather, it seems that image segmentation should affect the quality of the flanker representation as well.

In the present experiments, it appears that target attenuation (as reflected in a lower drift rate) slowed responding in the same-object (vs. different-object) condition. In line with this, the object-based CE modulations in Experiment 1 and Experiment 2 were accompanied by generally slower responses in the (near-)same than in the (near-)different condition. Interestingly, the main effect of object membership was not significant in the analysis of Kramer and Jacobson (1991; Experiment 3) – presumably due to insufficient statistical power. But slower responses in the same-object (vs. different-object) condition have often been found in experiments using other object membership manipulations, which has been attributed to an additional segmentation process needed in the same-object condition to separate the target from the flankers (Chen & Cave, 2006; Luo & Proctor, 2016; Shomstein & Yantis, 2002). Similarly, the target attenuation account can ascribe this speed difference to impaired access to task-relevant information when the target is hidden inside a higher-order perceptual object. This builds on findings from another research strand on global/local processing, which suggest weaker processing of local (vs. global) information (e.g., Miller, 1981; Navon, 1977; Palmer, 1978). For instance, Prinzmetal and Banks (1977) found worse target classification performance when the target formed a higher-level Gestalt with other items than when it did not.

The target attenuation account can also explain why prior research yielded a heterogeneous picture regarding the presence or absence of CE modulations by object manipulations that are not based on spacing or feature changes. A CE modulation was observed in several experiments (Chen & Cave, 2006, Experiment 2; Fox, 1998, Experiment 4; Luo & Proctor, 2016, Experiments 2 and 4A; Richard et al., 2008, Experiments 1–3), but in many others not (Chen & Cave, 2006, Experiment 1; Luo & Proctor, 2016, Experiments 1, 3, and 4B; Richard et al., 2008, Experiments 4–5; Shomstein & Yantis, 2002, Experiments 1–4). Specifically, most negative results have been found with stimulus material in which the target was part of a superordinate object in the same- but also in the different-object condition (Chen & Cave, 2006, Experiment 1; Luo & Proctor, 2016, Experiment 1; Richard et al., 2008, Experiments 4–5; Shomstein & Yantis, 2002, Experiments 1–4). Other experiments (Luo & Proctor, 2016, Experiments 3 and 4B; Richard et al., 2008, Experiments 4–5) that used different-object conditions, where the target was arguably still strongly grouped to the flankers, also yielded negative results. In contrast, almost all positive results were found when the target appeared detached in the different-object condition (Fox, 1998, Experiment 4; Luo & Proctor, 2016, Experiments 2 and 4A; Richard et al., 2008, Experiments 1–3). Taken together, one might speculate that an object manipulation only modulates the CE if it alters the perception of the target, which fits well with the target attenuation account. In particular, it is also consistent with the remarkable finding from Moore et al. (2021, Experiments 3b and 4b) that CEs are not affected by whether the target is grouped with flankers or neutral items. Note that other processes might very well be at play for the different stimulus materials used, which is unfortunately difficult to evaluate given the lack of more fine-grained analyses in previous studies.

Of course, selective attention can also be affected by changes in flanker processing. This seems to contribute to the space-based CE modulation, which was observed in Experiment 1, for instance. In a classic letter-based flanker task, Servant and Evans (2020) also found that the CE decreased with increasing flanker distance (0.4°, 1.0°, 2.5°). Because a CE was present for each distance (cf. Experiment 1), these data are well-suited to uncover specific effects of flanker distance via diffusion modeling. According to their DMC fits, smaller flanker distance not only decreased the drift rate of the target process but also increased the amplitude of distractor-based activation. Accordingly, both the distance and the object manipulation affect target processing strength, whereas only the distance manipulation affects flanker processing strength. This dissociation complicates the simple view that selective attention generally acts on object representations by highlighting the primacy of space in guiding selection processes.

Generally, the present modeling results and their interpretation are based on the dual-route framework implemented within the DMC. Thus, it might be useful to check whether other models, which to some extent rely on different theoretical assumptions, could also account for the observed empirical results and whether their application leads to the same theoretical conclusions. For example, one could consider extended versions of the DMC (e.g., with a time-varying drift rate for target processing; Richter et al., 2023), other evidence-accumulation models that share characteristics of dual-route frameworks (e.g., Heuer & Wühr, 2023; Hübner et al., 2010), models built within a single-process framework (e.g., the single-process spotlight model; White et al., 2011), or models other than evidence-accumulation models (e.g., two-state mixture models such as the activation-suppression race model; Miller & Schwarz, 2021).

In summary, the present study provides further evidence that a purely object-based mechanism is involved in human selective attention. It puts forward the idea that this object-based mechanism consists in a weakening of target processing strength when task-relevant information is hidden within a higher-order perceptual object. Methodologically, the presented work illustrates that diffusion modeling can advance theorizing by uncovering effects on latent variables that are more specific than manifest variables, such as RT.

## Footnotes

^1^
Intermediate values (9 ms, 1.6%) were obtained in a third object condition, in which flankers were neither connected to the target nor to the outer lines. Kramer and Jacobson (1991) employed this control condition to examine the gradedness of grouping effects. It is disregarded here.

^2^
In both the far-same and far-different conditions, CEs on mean RT, PC, and all nine RT deciles (see Figure 3) did not differ significantly from zero (all *p*s > .057).

^3^
For mean RTs, the object-based CE modulation was only significant for near (10 ms), *t*(39) = 2.40, *p* = .021, *d_z_* = 0.38, but not for far flankers (4 ms), *t*(39) = 1.12, *p* = .270, *d_z_* = 0.18. Similarly, for PCs, the object-based CE modulation was significant for near (2.2%), *t*(39) = 3.36, *p* = .002, *d_z_* = 0.53, but not for far flankers (0.6%), *t*(39) = 0.89, *p* = .377, *d_z_* = 0.14.

^4^
As the CE was never substantially above zero in the far conditions in Experiment 1, we primarily focused on the near conditions.

^5^
The same conclusions were reached when reiterating the same analyses for lines fitted only to the CEs at five percentiles (0.1, 0.3, …, 0.9) of the RT distribution.

^6^
Pilot modeling suggested that this general picture is robust to changes in the searched parameter space and in the minimized cost function (i.e., root-mean-square error instead of *G*^2^).

^7^
One well-known object-based account that cannot explain the CE modulation by object membership observed here under positional certainty is *attentional prioritization* (e.g., Shomstein & Yantis, 2002), which posits that target search starts within an already attended object and shifts to other objects only afterward. Since this restricts the scope of object-based selective attention to the strategic scanning order in search-like tasks (e.g., Egly et al., 1994), attentional prioritization is incompatible with the present data.

## Notes

**Publication Ethics:** All procedures performed in studies involving human participants were in accordance with the ethical standards of the institutional and/or national research committee and with the 1964 Declaration of Helsinki and its later amendments or comparable ethical standards. Informed consent was obtained from all individual participants included in the study.

**Authorship:** The last two authors share senior authorship.

**Open Data:** Raw data, model fits, analysis scripts, stimulus materials, and pre-registrations are available via the Open Science Framework at https://osf.io/js4h5/ (Experiment 1; Gierlich et al., 2023) and https://osf.io/pqdcz/ (Experiment 2; Mittelstädt et al., 2023). Experimental scripts are available via GitHub at https://github.com/igmmgi/jsPsychExperiments/tree/master/Experiments7%2B/flankerGrouping.

The pre-registered experiments and analyses were executed exactly as registered (https://osf.io/2pbfu, https://osf.io/x7svt).
